# Toxicity and efficacy of lenvatinib plus pembrolizumab in advanced endometrial cancer: a real-world retrospective analysis

**DOI:** 10.3389/fonc.2025.1622253

**Published:** 2025-09-08

**Authors:** Mali Barbi, Chung-Shien Lee, Husneara Rahman, Kit Ling Cheng, Veena S. John

**Affiliations:** ^1^ Division of Medical Oncology, Department of Medicine, Northwell Health Cancer Institute, New Hyde Park, NY, United States; ^2^ Cancer Center, Cold Spring Harbor Laboratory, Cold Spring Harbor, NY, United States; ^3^ College of Pharmacy and Health Sciences, St. John’s University, Queens, NY, United States; ^4^ Biostatistics Unit, Office of Academic Affairs, Northwell Health, New Hyde Park, NY, United States

**Keywords:** endometrial cancer, microsatellite-stable (MSS), KEYNOTE-775 trial, lenvatinib, pembrolizumab, dose optimization, adverse events

## Abstract

**Background:**

The treatment landscape for advanced recurrent endometrial cancer (EC) has been transformed with the introduction of lenvatinib and pembrolizumab, supported by results from the KEYNOTE-775 trial. However, the recommended 20 mg daily lenvatinib dose often results in significant toxicity, limiting its use in clinical practice.

**Objective:**

To evaluate the toxicity and efficacy of reduced (≤10 mg) versus higher (>10 mg) initial doses of lenvatinib combined with pembrolizumab in patients with advanced recurrent EC.

**Methods:**

In this retrospective cohort study, patients with EC treated with lenvatinib and pembrolizumab were stratified by initial lenvatinib dose into reduced (≤10 mg) and higher (>10 mg) groups. Study endpoints included progression-free survival (PFS), overall survival (OS) and treatment-related toxicity.

**Results:**

Of the 92 patients included, 62% initiated lenvatinib at ≤10 mg and only 14.1% received the recommended 20 mg dose. Baseline characteristics were comparable between groups, except for age (71.2 vs. 67.5 years; p = 0.003). Grade ≥2 adverse events occurred in 74% of patients, with half experiencing treatment interruptions, and 36% discontinuations, primarily due to fatigue, diarrhea, or thromboembolic events. While unadjusted PFS and OS did not differ significantly between groups (p = 0.074 and p = 0.148, respectively), age-adjusted analysis showed significantly higher hazard of progression or death in the reduced-dose group (HR: 2.92; 95% CI: 1.32–6.44; p = 0.008).

**Conclusion:**

This is the largest real-world study to date evaluating initial lenvatinib dosing strategies in advanced EC. Our findings suggest that although reduced starting doses (≤10 mg) are commonly used to mitigate toxicity, they may compromise efficacy. These results challenge current prescribing patterns and emphasize the need for prospective studies to define optimal dosing strategies.

## Introduction

Endometrial cancer (EC) is the most common gynecologic malignancy worldwide and the leading cause of gynecologic cancer-related death in the United States. Unlike many other cancers, EC survival rates have declined over the past four decades, highlighting an urgent need for better treatment strategies (1). The Cancer Genome Atlas (TCGA) has classified EC into four molecular subtypes: POLE-ultramutated, microsatellite instability-high (MSI-H)/mismatch repair deficiency (dMMR), copy-number low (p53 wild-type), and copy-number high (p53 mutant) (2). This classification has refined risk stratification facilitated molecularly tailored therapies, and guided clinical management (3). While platinum-based chemotherapy has long been the standard for advanced EC, immune checkpoint inhibitors (ICIs) have transformed treatment paradigms, particularly for MSI-H tumors. ICIs have shown high efficacy in MSI-H but limited activity in MSS/pMMR tumors (4–6). To address this limitation, combined chemo-immunotherapy strategies have been studied. The RUBY and NRG-GY018 trials demonstrated significantly improved PFS, leading to the adoption of chemo-immunotherapy as the preferred first-line approach for advanced EC (7, 8). Nevertheless, disease progression remains a significant challenge in pMMR tumors, highlighting the need for more effective second-line therapies (9, 10). Preclinical studies have shown that combining lenvatinib, a multikinase inhibitor, and pembrolizumab, a PD-1 inhibitor, yields synergistic antitumor activity beyond either agent alone (11). The safety and effectiveness of the combination were first assessed in Study 111/KEYNOTE-146 and later validated in the phase III KEYNOTE-775 trial, which demonstrated meaningful improvements in PFS and OS over chemotherapy. This led to FDA approval of pembrolizumab (200 mg every 3 weeks) in combination with lenvatinib (20 mg daily) for previously treated advanced EC (12, 13). However, toxicity remains a major barrier to sustained treatment. Frequently reported grade ≥3 adverse events (AEs) included diarrhea, hypertension (HTN), musculoskeletal disorders, fatigue, nausea, decreased appetite, vomiting, stomatitis, weight loss, proteinuria, dyspnea, cough, and rash (13, 14). In Study 111/KEYNOTE-146, approximately 66% of patients experienced grade ≥3 AEs; this increased to 88.9% in KEYNOTE-775, compared to 72.7% seen with chemotherapy. These toxicities led to dose reductions in two-thirds of patients, treatment interruptions in over 70%, and discontinuation in one-third of patients—primarily due to lenvatinib (12, 13). Although the recommended starting dose is 20 mg daily, the actual average administered doses were lower: 14.4 mg/day in Study 111/KEYNOTE-146 and 13.8 mg/day in KEYNOTE-775. Only 8.9% of patients were able to maintain the full dose for ≥ 6 months (12, 13), emphasizing the need for close monitoring and individualized dose adjustments (14, 15). Recognizing the high incidence of treatment-related toxicity in clinical trials, and the broader range of patients encountered in real-world settings, many clinicians have adopted lower starting doses of lenvatinib. To better understand the impact of this approach, we conducted a retrospective cohort study to assess the efficacy and tolerability of initiating lenvatinib at ≤10 mg versus >10 mg.

## Methods

### Patients and data collection

We conducted a retrospective cohort study of all patients with advanced recurrent EC treated with lenvatinib and pembrolizumab at Northwell Cancer Institute between September 2019 and January 2022. A total of 92 patients met eligibility criteria and were included in the analysis. Eligible patients were ≥18 years old, had histologically confirmed EC with progression after prior chemotherapy, and received at least three cycles of lenvatinib and pembrolizumab. Patients lost to follow-up without documentation of toxicity, response, or survival were excluded. Demographic and clinicopathologic data—including age, body mass index (BMI), race, ethnicity, Eastern Cooperative Oncology Group (ECOG) performance status, microsatellite instability status, PD-L1 expression, and prior treatment history—were obtained from the institution’s electronic medical record system. Treatment-related variables included lenvatinib starting dose, treatment duration, AEs necessitating dose modifications, treatment interruptions or discontinuations, disease progression, and survival outcomes. Disease progression or death was determined based on treating physician documentation, including clinical evaluations and imaging reports. This approach reflects the real-world nature of the dataset and aligns with retrospective methodology. All data were managed using REDCap (Research Electronic Data Capture) tools hosted at Northwell Health (16). The primary objective was to assess the efficacy of lenvatinib based on the starting dose of >10 mg (higher-dose group) vs. ≤10 mg (reduced-dose group) in terms of PFS and OS. Although the starting dose does not fully capture total drug exposure, it was used as a pragmatic surrogate, given its clinical relevance in guiding initial therapy, reflecting real-world prescribing patterns, and guiding clinical decision-making. A dichotomous classification (≤10 mg vs. >10 mg) was selected to mirror common clinical thresholds based on tolerability and patient characteristics. To contextualize treatment intensity, we also assessed treatment duration rates of dose reduction, interruption, and discontinuation across groups. Given the retrospective nature of this study, which involved the analysis of pre-existing medical records, direct patient involvement was not applicable. However, the insights gained from this study aim to inform and improve clinical practices, ultimately benefiting patients with advanced EC.

### Statistical analysis

Demographic and clinicopathologic characteristics were summarized using medians (interquartile range [IQR]), means (standard deviation [SD]), or proportions (n, %). PFS was defined as the time from treatment initiation to disease progression or death. Individuals who were alive without progression at their last follow-up time were considered censored. OS was defined as the time from treatment initiation to death from any cause, with patients remaining alive at their last follow-up, also censored. The Kaplan-Meier method was used to estimate one-year PFS and OS with 95% confidence intervals (CIs), and the log-rank test was performed to assess statistical significance between the dosing cohorts. To evaluate the association between lenvatinib starting dose and PFS, a multivariable Cox proportional hazards (PH) regression model was constructed. The initial model included starting dose, age, race, BMI, ECOG performance status, number of prior therapy lines, and MSI status. A backward elimination procedure was applied to derive the most parsimonious model. Hazard ratios (HRs) with 95% CIs were reported. Exploratory analyses were also conducted to evaluate the association between initial dose and toxicity outcomes, including treatment modifications and discontinuation.

## Results

### Demographic and clinicopathologic characteristics

Ninety-two patients met the inclusion criteria. The median follow-up was 8.6 months (range 3.87–17.17). The median age at treatment initiation was 70.0 years (IQR 65.5–76.4), and the median BMI was 28.5 kg/m² (IQR 23.9–34.7). Most patients (72.8%) had an ECOG performance status of 0–1, and 27.2% had an ECOG status of 2–3. The majority of patients were White (53.3%) or Black (33.7%). Most tumors were classified as pMMR (94.5%). PD-L1 expression ranged from 1–10% in 18.5% of patients, and was >10% in 4.3%. Initial lenvatinib dosing was clinician-guided based on age, performance status, and anticipated tolerability. Only 14.1% of patients-initiated therapy at 20 mg. Among those, 54% required reductions, mostly to 14 mg, due to toxicity. Starting doses were distributed as follows: 10 mg (38%), 8 mg (18.5%), 14 mg (15.2%), 12 mg (8.7%), and 4 mg (5.4%). Patients were stratified into reduced- (≤10 mg) and higher-dose (>10 mg) groups, comprising 62% and 38% of the cohort, respectively. Patients in the reduced-dose group were significantly older than those in the higher-dose group (71.2 vs. 67.5 years, p = 0.003). A higher proportion of ECOG 0–1 was observed in the higher-dose group (77% vs. 70%), though this was not statistically significant (p = 0.878). Serous histology predominated in both groups, followed by endometrioid. Carcinosarcoma and mixed histology were more common in the reduced-dose group, while clear cell carcinoma was more frequent in the higher-dose group (**Table 1**).

**Table 1:** Baseline characteristics by lenvatinib starting dose.

Characteristic	Lenvatinib ≤10 mg (n=57)	Lenvatinib >10 mg (n=35)	All Patients (n=92)	p-value
Age, years, median (IQR)	71.2 (67.5-78.2)	67.5 (63.6-73.0)	70.0 (65.5-76.4)	*0.003
BMI, median (IQR)	29.3 (24.9-33.2)	26.7 (23.4-37.8)	28.5 (23.9-34.7)	—
ECOG performance status, n (%) 0 1 2 3	23 (40.4%) 17 (29.8%) 15 (26.3%) 2 (3.5%)	17 (48.6%) 10 (28.6%) 7 (20.0%) 1 (2.9%	40 (43.5%) 27 (29.4%) 22 (23.9%) 3 (3.3%)	0.878
Race, n (%) White Black Asian Other	30 (52.6%) 20 (35.1%) 4 (7.0%) 3 (5.3%)	19 (54.3%) 11 (31.4%) 1 (2.9%) 4 (11.4%)	49 (53.3%) 31 (33.7%) 5 (5.4%) 7 (7.6%)	0.623
Ethnicity, n (%) Hispanic or Latino Not Hispanic or Latino Unknown	3 (5.3%) 54 (94.7%) 0 (0%)	1 (2.9%) 33 (94.3%) 1 (2.9%)	4 (4.4%) 87 (94.6%) 1 (1.1%)	0.562
Previous lines of therapy, n (%) 0 1 2 ≥ 3	2 (3.5%) 32 (56.1%) 18 (31.6%) 5 (8.8%)	0 (0%) 22 (62.9%) 11 (31.4%) 2 (5.7%)	2 (2.2%) 54 (58.7%) 29 (31.5%) 7 (7.6%)	0.814
Previously received chemotherapy, n (%)	55 (96.5%)	35 (100%)	90 (98%)	0.152
Presence of MMR deficiency, n (%)	5 (8.8%)	0 (0%)	5 (5.5%)	0.062
PDL1 Status, n (%) <1% 1-10% >10% Unknown	29 (50.9%) 9 (15.8%) 3 (5.3%) 16 (28.1%)	19 (54.3%) 8 (22.9%) 1 (2.9%) 7 (20.0%)	48 (52.2%) 17 (18.5%) 4 (4.3%) 23 (25.0%)	0.704
Histologic subtypes, n (%) Serous Endometrioid Carcinosarcoma Clear cell Mixed	31 (54.4%) 16 (28.1%) 6 (10.5%) 1 (1.8%) 3 (5.3%)	21 (60%) 8 (22.9%) 2 (5.7%) 3 (8.6%) 1 (2.9%)	52 (56.5%) 24 (26.1%) 8 (8.7%) 4 (4.3%) 4 (4.3%)	—
Baseline serum creatinine, median (IQR)	0.9 (0.7-1.2)	0.8 (0.7-1.0)	0.9 (0.7-1.1)	—
Baseline total bilirubin, median (IQR)	0.3 (0.2-0.4)	0.4 (0.3-0.5)	0.3 (0.2-0.4)	—
Lenvatinib starting dose 20 mg 14 mg 12 mg 10 mg 8 mg 4 mg			13 (14.1%) 14 (15.2%) 8 (8.7%) 35 (38.0%) 17 (18.5%) 5 (5.4%)	—

Data are n (%) unless otherwise indicated. Medians reported with IQR. — = not applicable. BMI, body mass index; ECOG PS, Eastern Cooperative Group performance status.

### Treatment-related toxicity

**Figure 1:**
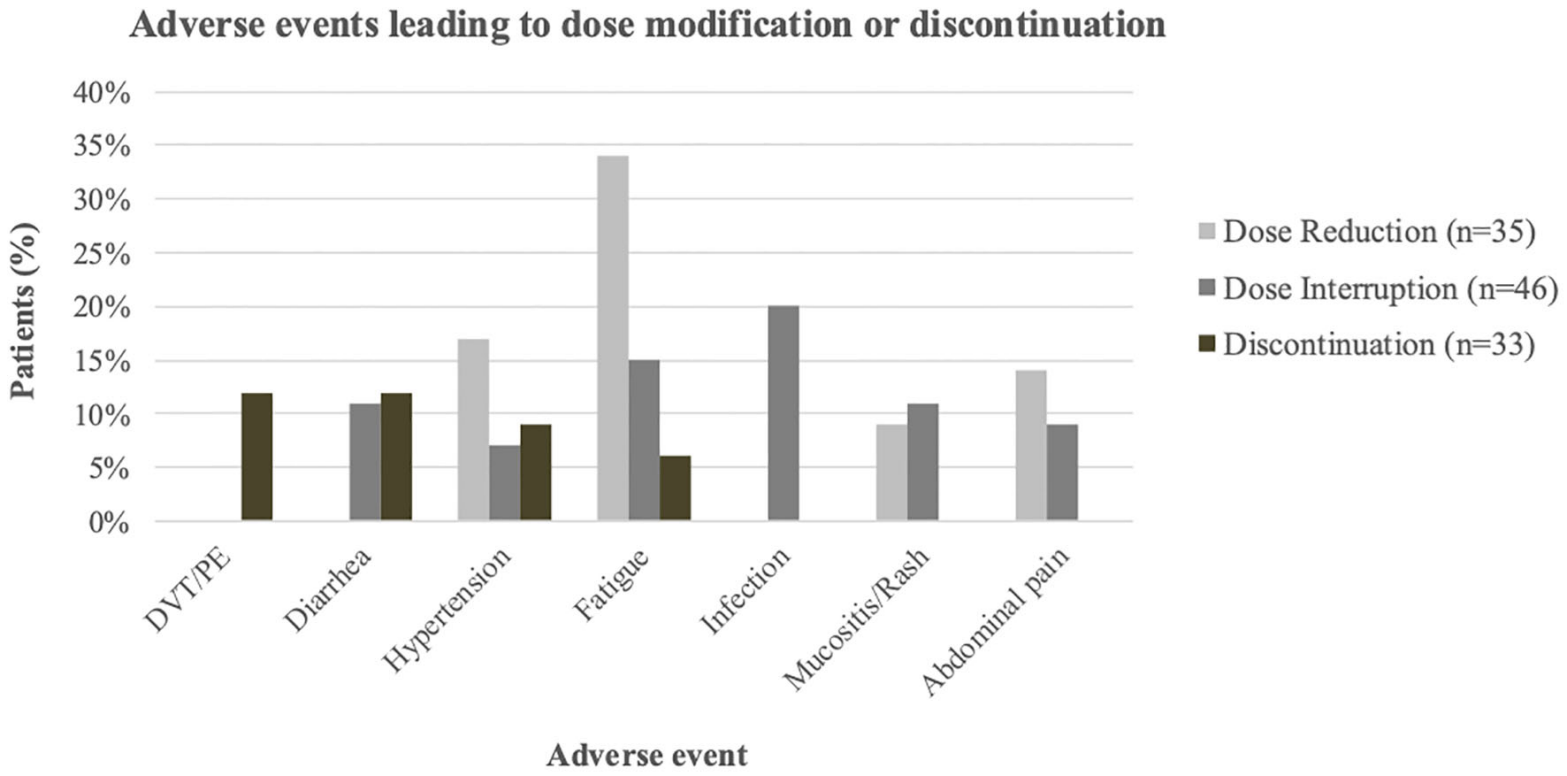
Most common Aes leading to lenvatinib dose modification or discontinuation. Grouped bars show the percentage of patients with dose reduction (light gray), dose interruption (dark gray), or discontinuation (brown); Events occurring in fewer than two patients per category are not shown.

Grade ≥2 AEs were reported in 74% of patients. Dose reductions were more frequent in the reduced-dose group (46%) than in the higher-dose group (37%). Treatment interruptions occurred in approximately half of patients in both groups (51% vs. 49%). The median time to first interruption and to permanent discontinuation were both longer in the higher-dose group (65 vs. 28.5 days, and 120 vs. 74 days, respectively). Infections were the most common cause of interruption, occurring more frequently in the higher-dose group (24% vs. 17%). Intolerable fatigue was a leading cause of both interruption and dose reduction. The most frequent reasons for permanent treatment discontinuation were thromboembolic events (DVT/PE), diarrhea, and HTN (**Figure 1**, **Table 2**).

**Table 2:** Treatment-related toxicity.

Toxicity measure	Lenvatinib ≤10 mg (n=57)	Lenvatinib >10 mg (n=35)	All patients (n=92)
Treatment related toxicity, n (%)	42 (74%)	26 (74%)	68 (74%)
Dose reduction, n (%)	26 (46%)	13 (37%)	39 (42%)
Treatment discontinuation, n (%)	21 (37%)	12 (34%)	33 (36%)
Dose interruption, n (%)	29 (51%)	17 (49%)	46 (50%)
Median time to dose interruption, days (IQR)	28.5 (20–69.5)	65.0 (43.3–97.8)	46.5 (20–94.0)
Median time to treatment discontinuation, days (IQR)	74 (49–168)	120 (55–241)	84 (50–199)

### Clinical efficacy and survival

**Figure 2:**
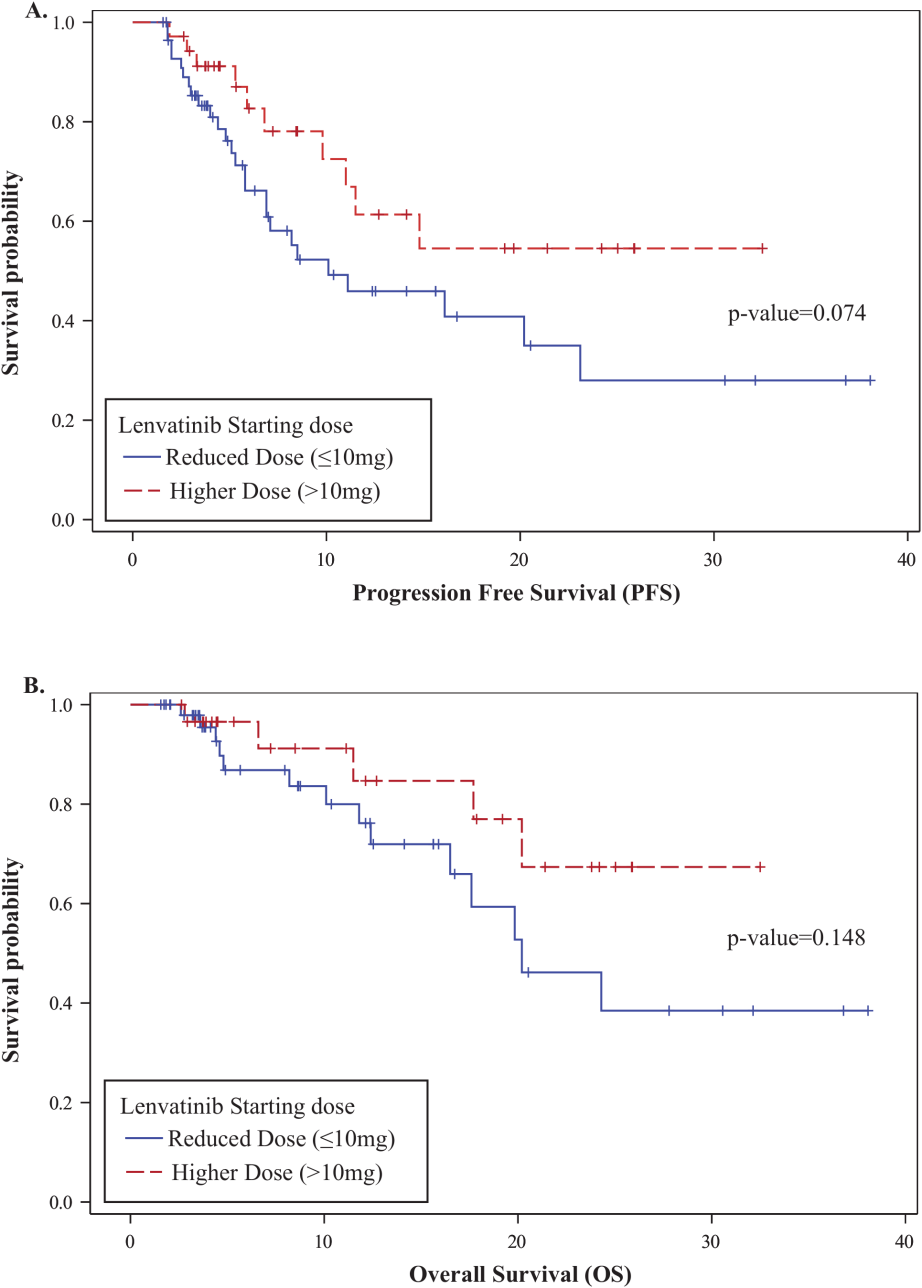
Kaplan-Meier survival analysis by lenvatinib starting dose. **(A)** Progression-Free Survival (PFS): One-year PFS was 0.61 (95% CI: 0.37–0.78) in the higher-dose group and 0.46 (95% CI: 0.30–0.61) in the reduced-dose group (p = 0.074). **(B)** Overall Survival (OS): One-year OS was 0.85 (95% CI: 0.58–0.95) in the higher-dose group and 0.76 (95% CI: 0.57–0.88) in the reduced-dose group (p = 0.148).

**Figure 3:**
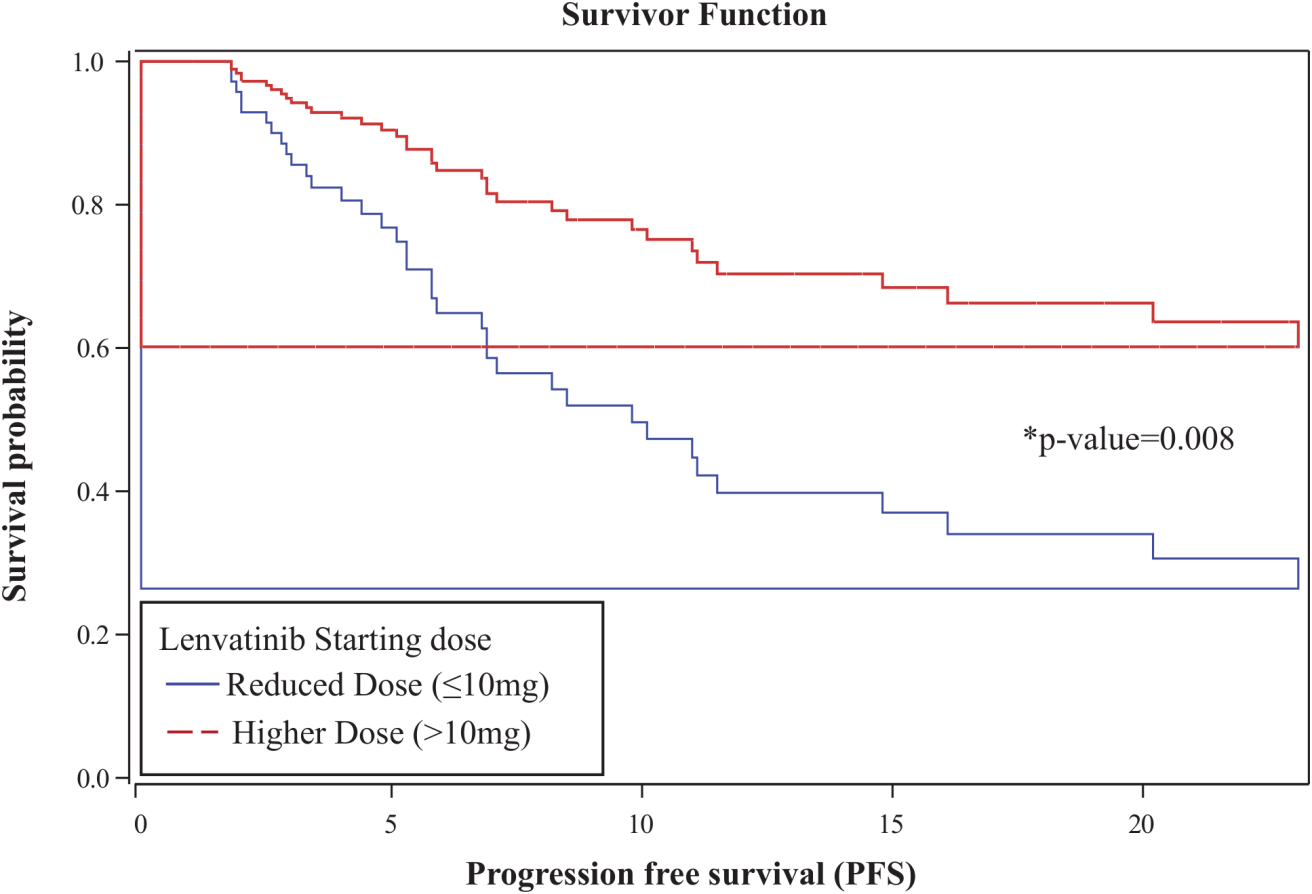
Age-adjusted Cox proportional hazards analysis for progression-free survival. Patients in the reduced-dose group (≤10 mg) had a significantly higher hazard of progression or death compared to those in the higher-dose group (>10 mg) (HR: 2.92; 95% CI: 1.32–6.44; p = 0.008).

The overall one-year PFS rate was 0.52 (95% CI: 0.38–0.64), and the one-year OS rate was 0.79 (95% CI: 0.65–0.88). No statistically significant differences in PFS or OS were observed between the higher- and reduced-dose groups: one-year PFS was 0.61 vs. 0.46 (p = 0.074), and OS was 0.85 vs. 0.76 (p = 0.148), respectively (**Figure 2**). In a multivariable Cox proportional hazards analysis including starting dose, age, BMI, race, ECOG, number of prior therapy lines, and MSI status, only age and starting dose remained in the final model following backward elimination. After adjusting for age, patients in the reduced-dose group (≤10 mg) had a significantly higher hazard of progression or death compared to those in the higher-dose group (>10 mg) (HR: 2.92, 95% CI: 1.32–6.44; p = 0.008) (**Figure 3**, **Table 3**).

**Table 3:** Cox proportional hazards regression model: maximum likelihood estimates.

Parameter	DF	Estimate	Std. error	Chi-square	P-value	Hazard ratio	95% CI (Lower–Upper)
Start dose ≤10 mg	1	1.070	0.404	6.998	0.008	2.92	1.32 – 6.44
Age at start of lenvatinib	1	-0.052	0.020	6.639	0.010	0.95	0.91 – 0.99

## Discussion

The KEYNOTE-775 trial established lenvatinib and pembrolizumab as the standard of care for patients with pMMR advanced recurrent EC (12, 13). However, the recommended 20 mg daily dose of lenvatinib presents significant toxicity challenges in clinical practice (12, 14, 17), often requiring empirical dose reductions to improve patient tolerability. This concern is further illustrated by a Korean multicenter retrospective study, in which 83% of patients initiated lenvatinib at 20 mg but 56.2% required subsequent dose reductions due to toxicity, highlighting widespread tolerability issues. Notably, ORR and PFS in that cohort were lower than those reported in KEYNOTE-775, potentially reflecting genetic and demographic differences between Korean and Western populations, and underscoring the influence of ethnicity on treatment outcomes (18). Given these challenges, our real-world retrospective study evaluated whether initiating lenvatinib at a reduced dose (≤10 mg) could improve tolerability without compromising efficacy. Compared to our cohort, which reflected broader clinical diversity and individualized, physician-guided dosing strategies, the KEYNOTE-775 trial enrolled a more homogeneous population—characterized by better baseline performance status, fewer aggressive histologic subtypes, and universal initiation at 20 mg lenvatinib. In our real-world cohort, 62% of patients started treatment at ≤10 mg and only 14.1% at 20 mg. Our cohort also included a higher proportion of patients with ECOG ≥2 and aggressive histologic subtypes, including carcinosarcoma. Although unadjusted comparisons showed no statistically significant differences in PFS or OS between dose groups, age-adjusted analysis revealed a significantly higher risk of progression or death with initial dosing at ≤10 mg (HR: 2.92; p = 0.008). Notably, only 34% of patients in the higher-dose group received the full 20 mg, with most starting at intermediate doses, suggesting that intermediate starting doses may better balance efficacy and tolerability. Our findings align with those of How et al. (19), who observed no significant differences in baseline characteristics or survival outcomes (PFS or OS) between patients who received a reduced starting dose—most commonly 14 mg—and those who received the recommended 20 mg dose. In contrast, Zammarrelli et al. (20), reported shorter PFS in patients initiating treatment at 20 mg. These contrasting findings may reflect variability in clinical practice patterns and patient populations. Notably, our cohort had a higher proportion of ECOG 0-1 patients (43.5% vs. 28% in Zammarrelli et al) which may have contributed to more favorable outcomes. Additionally, initiating lenvatinib at 20 mg with subsequent dose reductions could lead to higher rates of treatment interruptions, potentially limiting the sustained therapeutic synergy between pembrolizumab and lenvatinib. Dose optimization has been observed in other malignancies, such as hepatocellular carcinoma, where the FDA-approved lenvatinib dose ranges from 8 to 12 mg daily (21). Our study has several limitations, including its retrospective design, which carries the risk of incomplete data capture and selection bias, and the lack of MSI-H subgroup analysis due to the small sample size. To enhance statistical reliability and align with real-world prescribing patterns, we used a dichotomous dose classification (≤10 mg vs. >10 mg), which may have masked finer dose-response patterns.

In conclusion, this is the largest real-world evaluation of the lenvatinib–pembrolizumab combination in advanced EC to date, offering important insights into the impact of initial dosing strategies on patient outcomes. We observed a clear trend to initiate lenvatinib at a reduced dose, with only 14.1% of patients receiving the full 20 mg, regardless of ECOG performance status. Clinicians should carefully weigh the risks and benefits of various lenvatinib starting doses. Our findings suggest that intermediate starting doses may provide a more favorable balance between efficacy and tolerability. Prospective, controlled studies are needed to determine the optimal lenvatinib starting dose that balances efficacy and tolerability in patients with pMMR advanced EC.

## Data Availability

Aggregated data underlying the results are included in the article and its Supplementary Material. Because the study involves patient-level clinical information, the underlying individual-level data are not publicly available due to privacy and institutional restrictions. De-identified data may be provided by the corresponding author upon reasonable request and subject to required approvals (IRB# 22-0924) and a data use agreement.
